# Supplementary material for: Large-scale 2D heterostructures from hydrogen-bonded organic frameworks and graphene with distinct Dirac and flat bands
Source: Nat Commun. 2024 Jul 15;15:5934. doi: 10.1038/s41467-024-50211-5 (PMC11250822; doi:10.1038/s41467-024-50211-5)
Supplement: Supplementary file 1 — Supplementary Information [file 41467_2024_50211_MOESM1_ESM.pdf]

Supplementary information for

Large-scale 2D heterostructures from hydrogen-bonded organic frameworks and graphene with distinct Dirac and flat bands

Xin Zhang, Xiaoyin Li, Zhengwang Cheng, Aixi Chen, Pengdong Wang, Xingyue Wang, Xiaoxu Lei, Qi Bian, Shaojian Li, Bingkai Yuan, Jianzhi Gao, Fang-Sen Li, Minghu Pan and Feng Liu

Contents

Supplementary Notes

Note 1. Atomic structure and bands of the THPB-HOF/HOPG heterostructure……….3

Note 2. Large scaled self-assembled THPB monolayer/graphite heterostructure.........6

Note 3. STM imaging with different biases……………….......................................7

Note 4. The chirality of H-bond hollow rings……………………………………........7

Note 5. ARPES E-k intensity plot and two CECs of bare HOPG surface....................8

Note 6. The carrier velocity of Dirac bands in THPB-HOF…………………..….….....10

Note 7. Local DOS mapping at two characteristic energies on THPB-HOF…………..11

Note 8. Predicted AFM insulator of THPB-HOF…………………………………….....13

Note 9. Similar THPB framework on MOS_2 substrate……………………………………15

Supplementary Figures and Tables

Fig. 1. THPB-HOF/graphene heterostructure without structural optimization.............3

Fig. 2. THPB-HOF/graphene heterostructure after structural optimization..................4

Fig. 3. Calculated band structure along Γ-K path………………………......................5

Fig. 4. STM image of large-scale THPB monolayer/graphene heterostructure...........6

Fig. 5. STM images with different biases…………………………............................7

Fig. 6. The chirality of H-bond hollow-rings……..8

Fig. 7. ARPES E-k intensity plot and two CECs of bare HOPG surface…….............9

Fig. 8. The linear fitting of Dirac bands near the band crossing point..........................10

Fig. 9. Differential conductance maps measured on THPB-HOF……….......................11

Fig. 10. Theoretical prediction of AFM state……………………………………..……13

Fig. 11. Large-scale STM image of THPB molecular island formed on MoS2 clean surface……………………………………………………………………………………15

Fig. 12. High resolution STM images and dI/dV tunneling spectroscopic measurements on THPB on MoS_2……………………………………………………..……..……16

Fig. 13. The optical image of HOPG sample……………………....…...……….…………17

Fig. 14. Structural relaxation of the THPB-HOF monolayer on top of the eight-layered HOPG substrate……………………………………………………………..……..18

Fig. 15. In-situ room-temperature Raman spectrum……………………….……...…19

Fig. 16. ARPES observation of the THPB/BLG-SiC bands………………..………..20

Supplementary References……………..……..…………………………………..21

Supplementary Results and Discussions

Supplementary Note 1. Atomic structure and bands of the THPB-HOF/HOPG heterostructure

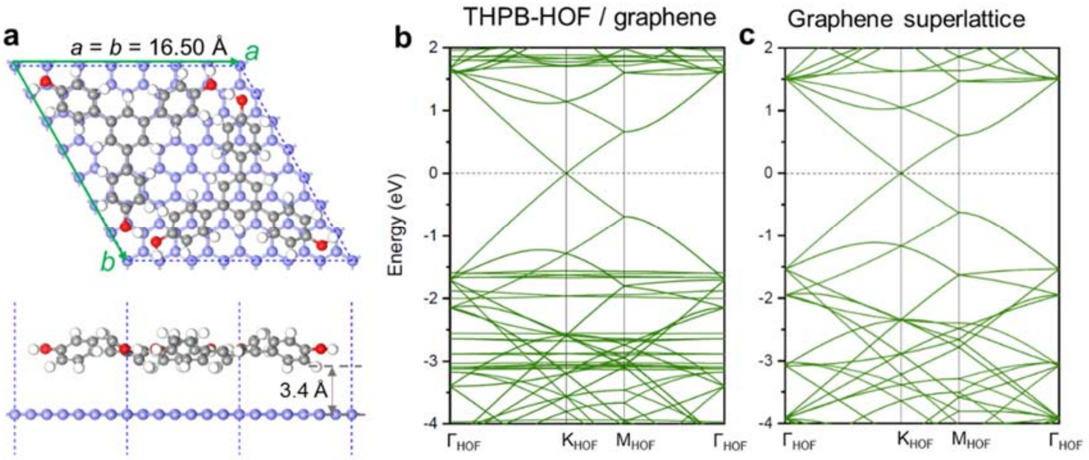

Supplementary Fig. 1 l THPB-HOF/graphene heterostructure without structural optimization. a, The atomic structure of THPB-HOF/graphene. Here the lattice constants of THPB-HOF follow the experimental values and the graphene supercell is uniformally compressed to fit into the unit cell of THPB-HOF. The distance from phenyl ring to the substrate is set to 3.4 Å. **b-c**, the calculated band structures of THPB-HOF/graphene (**b**) and graphene superlattice without THPB-HOF (**c**). Since the THPB-HOF monolayer and graphene are stacked together without performing structural optimization, the band structure of each individual miantains intact.

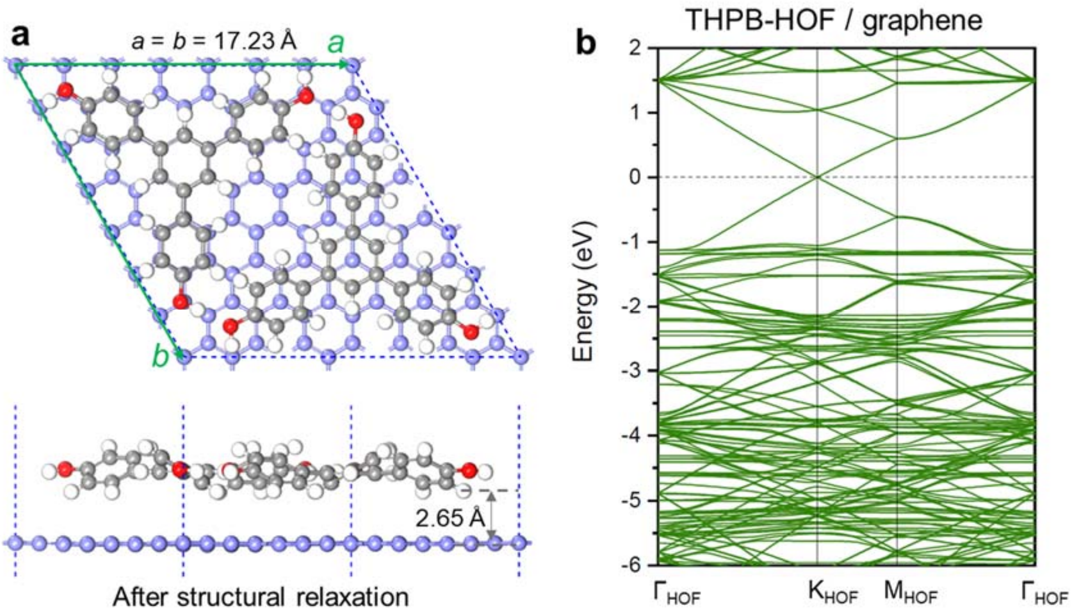

Supplementary Fig. 2 | THPB-HOF/graphene heterostructure after structural optimization. a, The atomic structure of optimized THPB-HOF/graphene. The optimized lattice constant and interlayer distance are 17.23 and 2.65 Å, respectively. Due to the interaction from the substrate, three corner phenyl rings tend to tilt instead of parallel to the substrate. **b**, the calculated band structure of THPB-HOF/graphene.

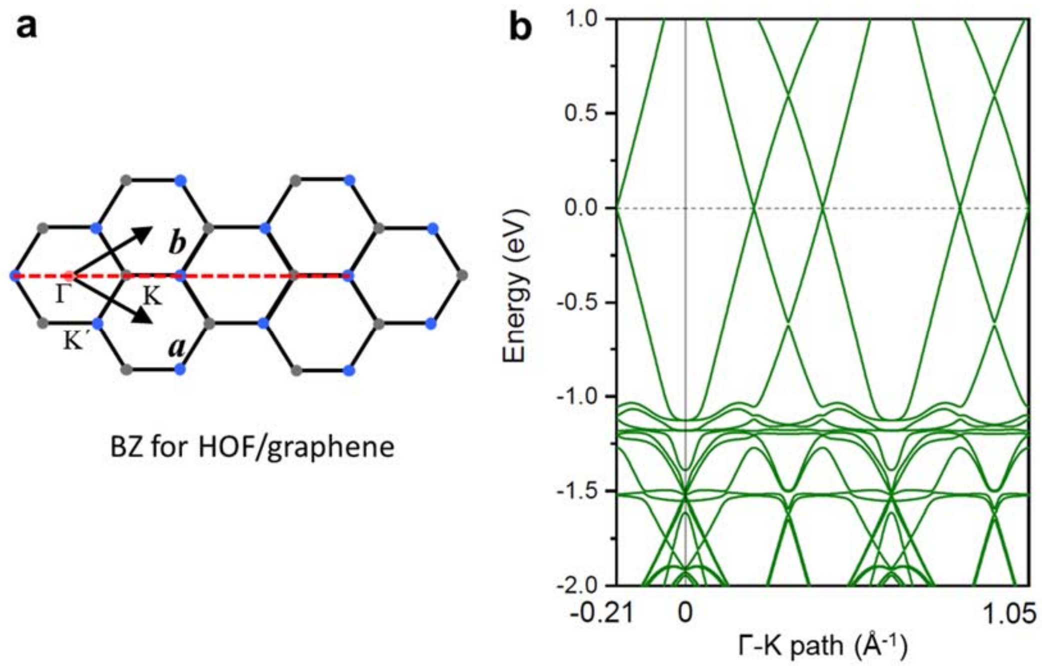

Supplementary Fig. 3 | Calculated band structure along Γ-K path. a, Schematic drawing of BZ of THPB-HOF. Red dashed line indicates the k path of calculated band structure in (**b**).

Supplementary Note 2. Large scaled self-assembled THPB monolayer/graphite heterostructure

A nearly-full coverage of THPB-HOF is assemblied on HOPG substrate. **Suppl. Fig. 4a** shows a large-scale uniformity and long-range order of HOF on the surface in the scan range of 125×125 nm^2. Besides, there are three domains observed. By zooming into a small area, these domains display same orientation, aligned with slightly shift of each HOF lattice (**Suppl. Fig. 4b**). The mesoscale ordered 2D THPB-HOF has enabled the ARPES characterization.

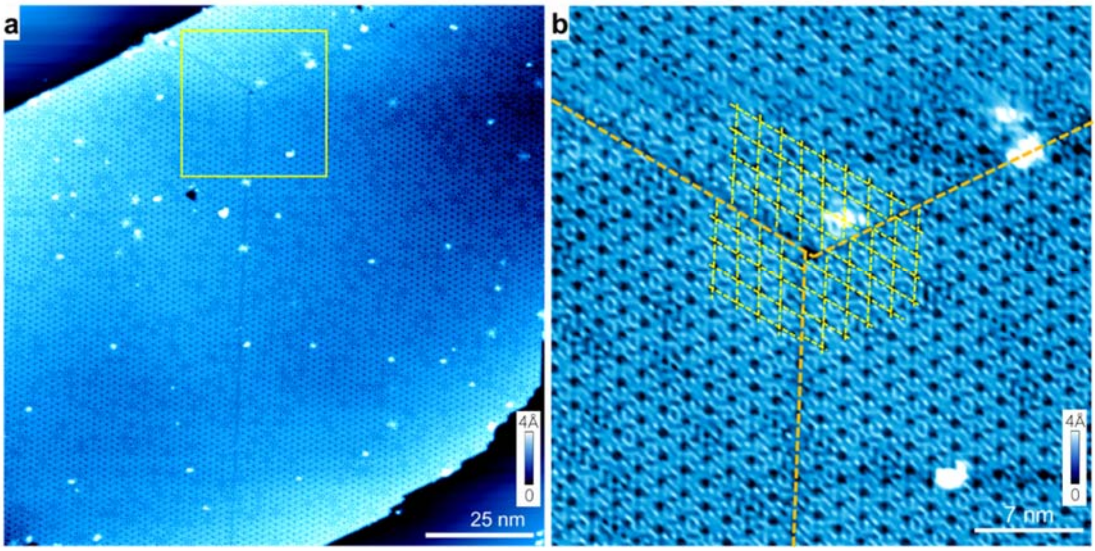

Supplementary Fig. 4. STM image of large scale self-assembled THPB monolayer/graphene heterostructure. **a**, STM image of nearly-full coverage of THPB-HOF. The image size is 125×125 nm^2 with the setting parameters of V_B=-2.0 V and I_T=10 pA. **b**, zoom-in image showing three domains with domain boundaries, taken at the region marked with yellow square in (**a**). The image size is 35×35 nm^2. The orange dashed lines indicate the domain boundaries and yellow dashed lines display the HOF lattices in each domain.

Supplementary Note 3. STM imaging with different biases

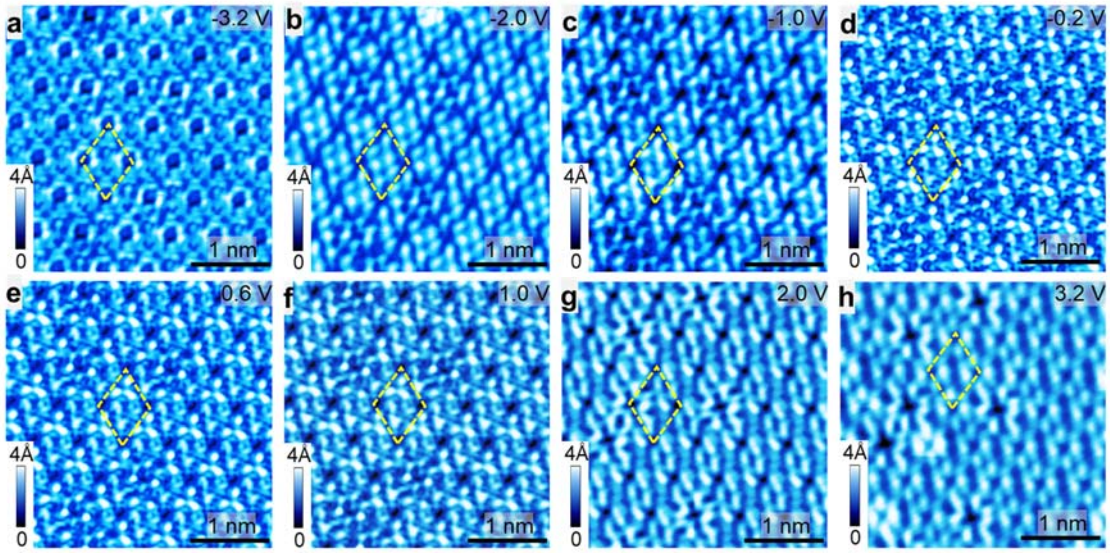

Supplementary Fig. 5 | STM images with different biases. a-h, A series of STM images taken with different biases from -3.2 V to +3.2 V.

Supplementary Note 4. The chirality of H-bond hollow-rings

In STM imaging of THPB-HOF, we observed two different kinds of patterns for H-bond hollow-ring, as shown in **Suppl. Fig. 6a** and **6b**. By comparing to the models (**Suppl. Fig. 6c**) with considering the clockwise and anticlockwise rotation of six hydrogen-bonds surrounding the hollow site, two types of H-bond hollow-rings agree well with the left hand and right hand of chirality of six hydrogen-bonds rotation. According to Ref. [1], the low-barrier hydrogen bond can easily switch the direction between O…H-O and O-H…O. We further did the statistical analysis by counting the numbers of the left and right chirality from many topographic STM images, the percentages of the left and right chirality are about 21% and 79%, respectively.

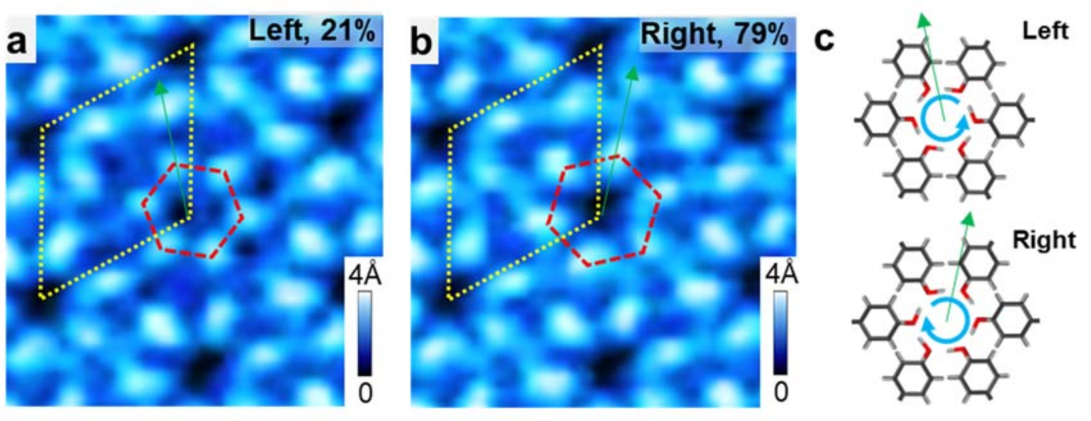

Supplementary Fig. 6 I The chirality of H-bond hollow-rings. a-b, STM images for the anticlockwise (left) and clockwise (right) rotation of six hydrogen-bonds surrounding the hollow site. **c,** the models illustrating the left- and right- hand rotations.

Supplementary Note 5. ARPES E-k intensity plot and two CECs of bare HOPG surface

In the energy range from 0 to -6.8 eV and the momentum range of ±0.6 Å^{-1}, three major band features are observed, including one non-dispersive band at -2.9 eV, and two dispersive bands at -4.0 eV around Γ point. The feature at -2.9 eV could be attributed to the localized state of isolated carbon atoms, surface or grain boundaries. Lanzara *et al.* [2] observed such non-dispersive features in HOPG at -2.9 eV, -4.3 eV and -7.8 eV, and suggested their origin be the non-k-conserving transitions or elastic electron scattering induced by inhomogeneity or disorder. For the two dispersive bands extending towards higher binding energies, earlier ARPES [3-5] and band calculations [6-9] have identified them to be the σ bands, *i.e.*, the upper σ_1 and lower σ_2 band. Besides, there are two valence π bands (π_1 and π_2), locate at -8.3 eV and -6.4 eV at Γ point and disperse strongly towards the E_F near the K point of the Brillouin zone (BZ), which are out of the scope of our ARPES and cannot be observed. Overall, in the ranges of 0~-2.0 eV and ±0.6 Å^{-1}, the bare HOPG surface has no band, to provide a clean background for observing the THPB-HOF bands.

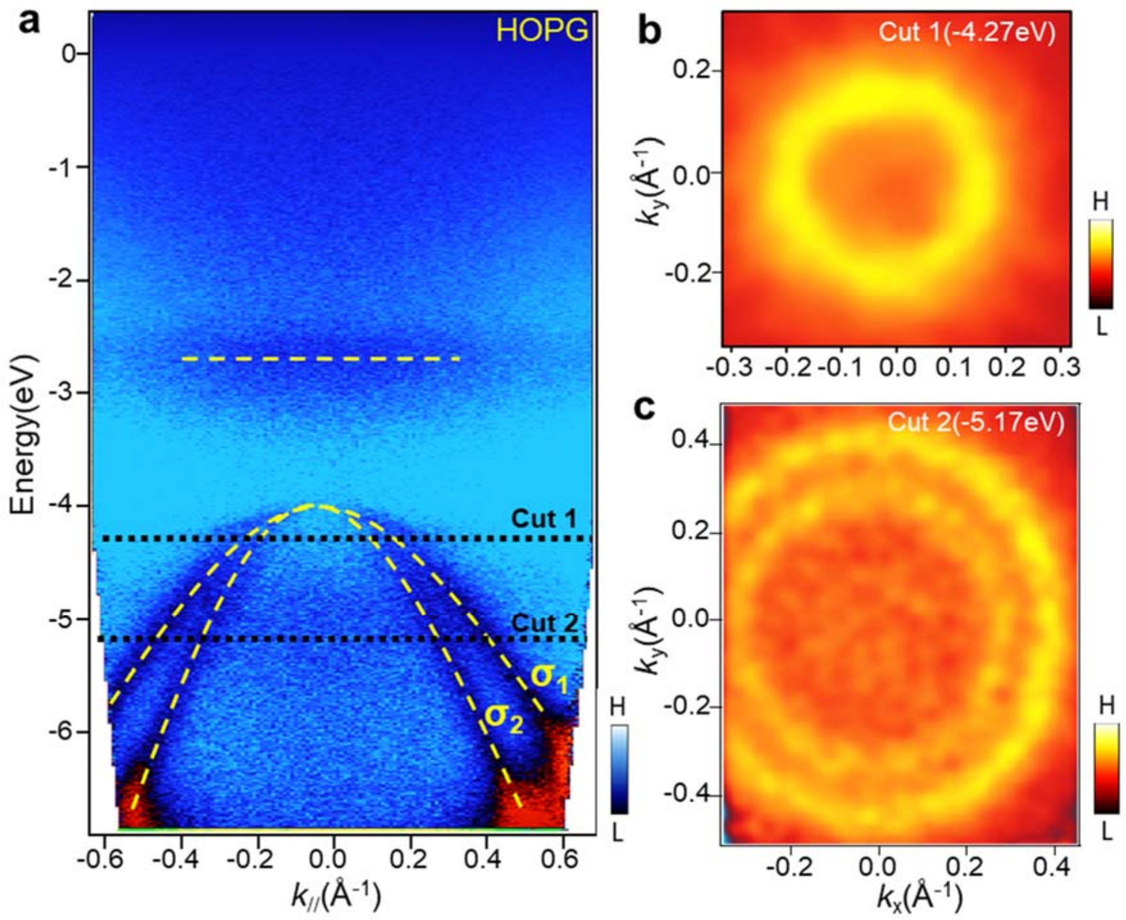

Supplementary Fig. 7 | ARPES E-k intensity plot and two CECs of bare HOPG surface. a, The photoemission intensity plot of E_B *vs.* k along the K_{HOPG}-Γ-K_{HOPG} direction at k_y=0 taken from the bare HOPG surface. K_{HOPG} is the K point of HOPG surface BZ. Yellow dahed lines indicate the energies of two CECs in **(b)** and **(c).** The CECs of σ_1 and σ_2 bands in HOPG show as concentric circles for the reason that HOPG has in-plane mosaic structure. Note, these σ bands of HOPG are about 4 eV below the $E_{F.}$

Supplementary Note 6. The carrier velocity of Dirac bands in THPB-HOF

The carrier velocity of Dirac bands can be obtained by linear fitting the DFT bands near the band crossing point. After fitting the two branches of Dirac bands as shown in **Suppl. Fig. 8**, we obtain the corresponding carrier velocities to be 0.226 and 0.277 eV·Å respectively. For brevity, we use an average value of 0.252 eVÅ as the carrier velocity of Dirac bands in THPB-HOF.

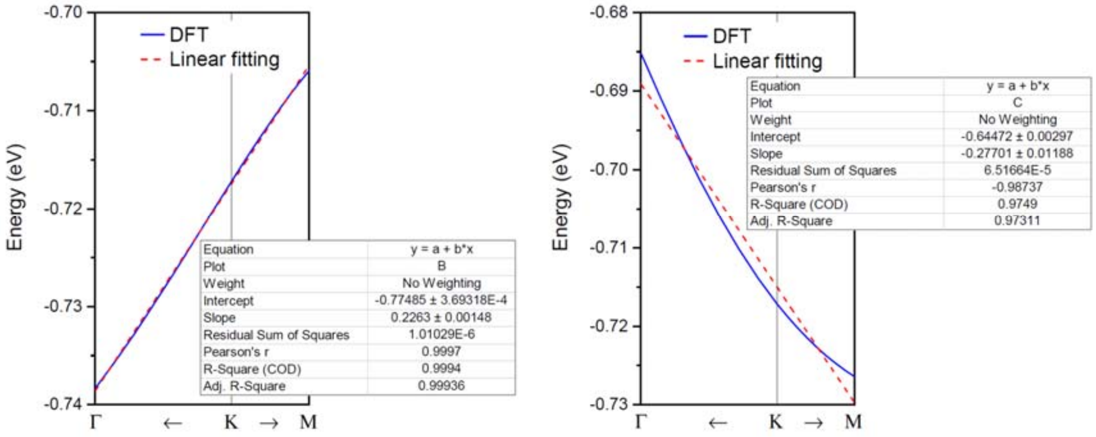

Supplementary Fig. 8 | The linear fitting of Dirac bands near the band crossing point. The fitting slope is the corresponding carrier velocity in unit of eVÅ.

Supplementary Note 7. Local DOS mapping at two characteristic energies on THPB-HOF

To visualize the distribution of such magnetic state in real space, we carried out the differential conductance mapping on THPB-HOF monolayer. Local DOS mappings are taken at two characteristic energies, *e.g.* -300 meV (occupied states) and +27 meV (the peak energy), as shown in **Suppl. Figs. 9a** and **9b**, respectively. The white-dashed lines denoted the unit cells and THPB model is overlaid with dI/dV maps for eyes-guided. Both of maps show the LDOS difference between "faulted halves" and "unfaulted halves", indicating the interaction between the HOPG substrate and THPB monolayer. Second, multiple inter/intra-molecular conducting channels appear, indicating the carriers hopping *via* two different routes, *e.g.* the H-bonds mediated by O/H atoms and the covalent bonds mediated by benzenes in THPB. Interestingly, for the LDOS map at +27 meV, the LDOS of magnetic states is more prominent at the THPB molecule in "unfaulted half" and the edges of THPB molecule in "faulted half" than the elsewhere, manifesting the internal spatial distribution of spin states for molecules.

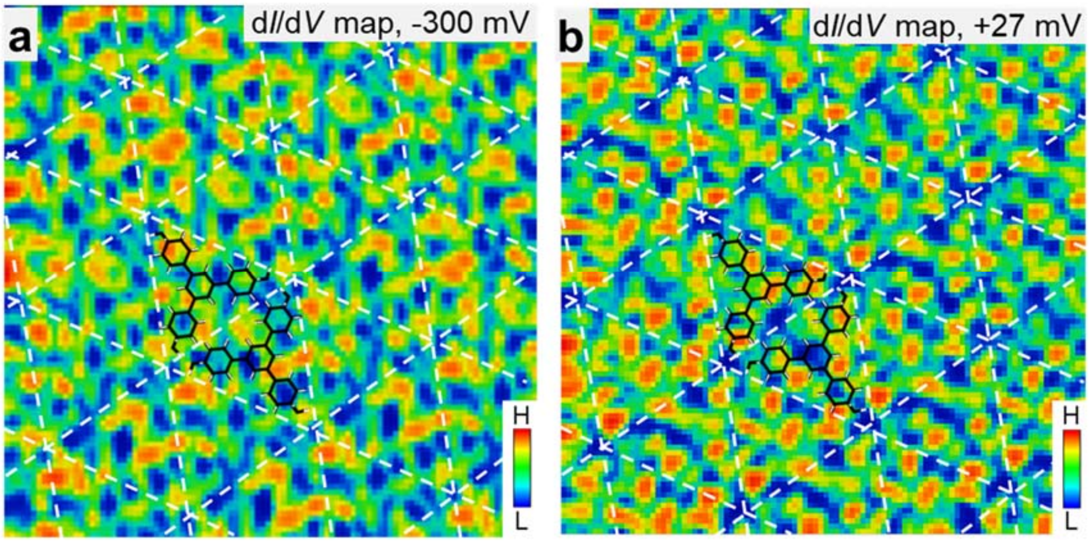

Supplementary Fig. 9 | Differential conductance maps measured on THPB-HOF. a-b, Differential conductance images (dI/dV mapping) taken simultaneously with the biases of -300 meV and +27 meV, respectively. The white dashed lines and the THPB

model is overlapped with the images for eyes-guided. All dI/dV spectra were measured with V_B=0.3 V, I_T=200 pA and a bias modulation of 7 mV at the temperature of 77 K.

Supplementary Note 8. Predicted AFM insulator of THPB-HOF

When the dual topological flat bands of THPB-HOF are half-filled, we confirm an antiferromagnetic (AFM) ground state and the electronic bands change from a nonmagnetic metal to an AFM insulator (**Suppl. Fig. 10**), indicating possible Mott insulating state. To confirm it, we estimate the onsite electron repulsion U of the hole-doped THPB-HOF (only the condition of half filling is considered) by employing a tight-binding (TB) model plus the onsite Coulomb interaction. The model Hamiltonian reads as:

$$H = \sum_{\langle ij \rangle \alpha} -t_{ij}(c_{i\alpha}^{\dagger} c_{j\alpha} + c_{j\alpha}^{\dagger} c_{i\alpha}) + \sum_i U n_{i\uparrow} n_{i\downarrow}. \quad (1)$$

The first term is the nearest-neighbor (NN) hopping, and the second term is the onsite Coulomb interaction. $c_{i\alpha}^{\dagger}$ and $c_{i\alpha}$ are the electron creation and annihilation operators on site *i* of spin *α*. $n_{i\alpha} = c_{i\alpha}^{\dagger} c_{i\alpha}$ is the spin-density operator. Given that antiferromagnetic (AFM) state is spontaneously generated by the onsite electron repulsion under this condition, we can use a simplified *t*-*J* model to replace Suppl. Eq. (1) [[10]]:

$$H = \sum_{\langle ij \rangle \alpha} -t_{ij}(c_{i\alpha}^{\dagger} c_{j\alpha} + c_{j\alpha}^{\dagger} c_{i\alpha}) + \sum_i (-1)^i \lambda_M c_i^{\dagger} s^z c_i. \quad (2)$$

Here the second term represents the AFM order driven by the onsite electron repulsion, therefore λ_M and U are related. Within the mean-field approximation, λ_M = (*m*/2)U and $m = \langle n_{i\uparrow} - n_{i\downarrow} \rangle$ representing the spontaneous magnetic moment. For the hole-doped THPB-HOF we have *m* = 2 as illustrated in **Suppl. Fig. 10a**. By fitting DFT bands using Suppl. Eq. (2), we obtain the fitting parameters of *t* = 0.023 and λ_M = *U* = 0.12 eV, and a *U*/*t* value of 5.22. The fitting results are shown in **Suppl. Fig. 10c**.

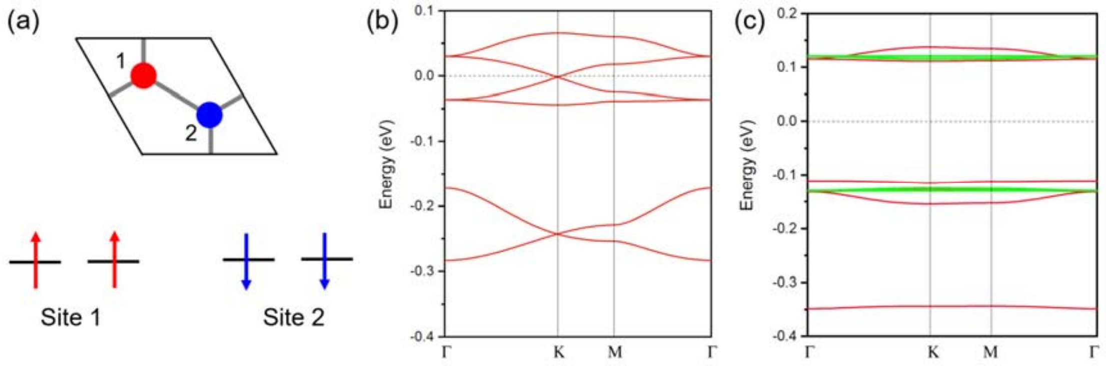

Supplementary Fig. 10 | Theoretical prediction of AFM state. a, The schematic diagram of the AFM model in the hexagonal lattice. Each unit cell contains two sites, and each site has two orbitals (p_x and p_y) as shown in the top panel. The bottom panel illustrates the state-filling scheme when the model system is half-filled. The red and blue arrows indicate the spin up and down states. **b,** The band structures of nonmagnetic state. **c,** The band structures of the AFM ground state. The red and green represent DFT and model fitting results respectively.

Supplementary Note 9. Similar THPB framework on MOS_2 substrate

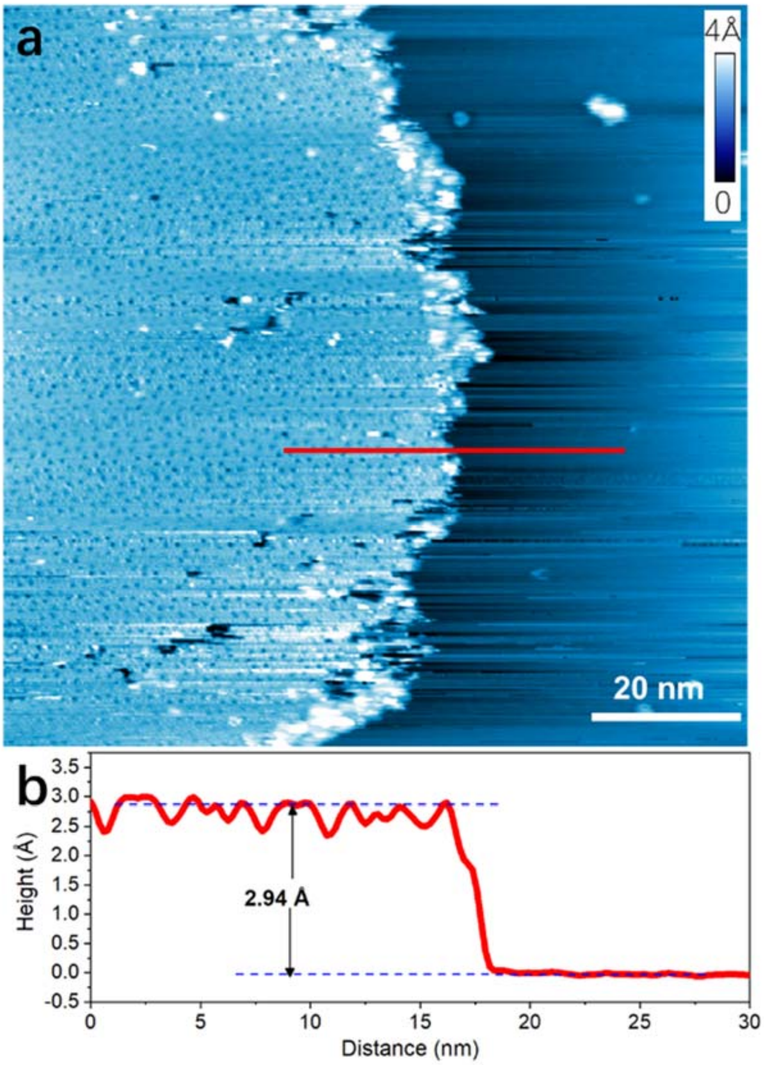

Supplementary Fig. 11 | Large-scale STM image of THPB molecular island formed on MoS_2 clean surface. a, THPB island formed on vacuum-cleaved MoS_2 surface. The image is 100×100 nm^2 with the setting parameters of V_B :-5V and I_T:5pA. **b**, the height profile measured along the red line in panel **a**, which shows the thickness of monolayer THPB is about 2.94 Å.

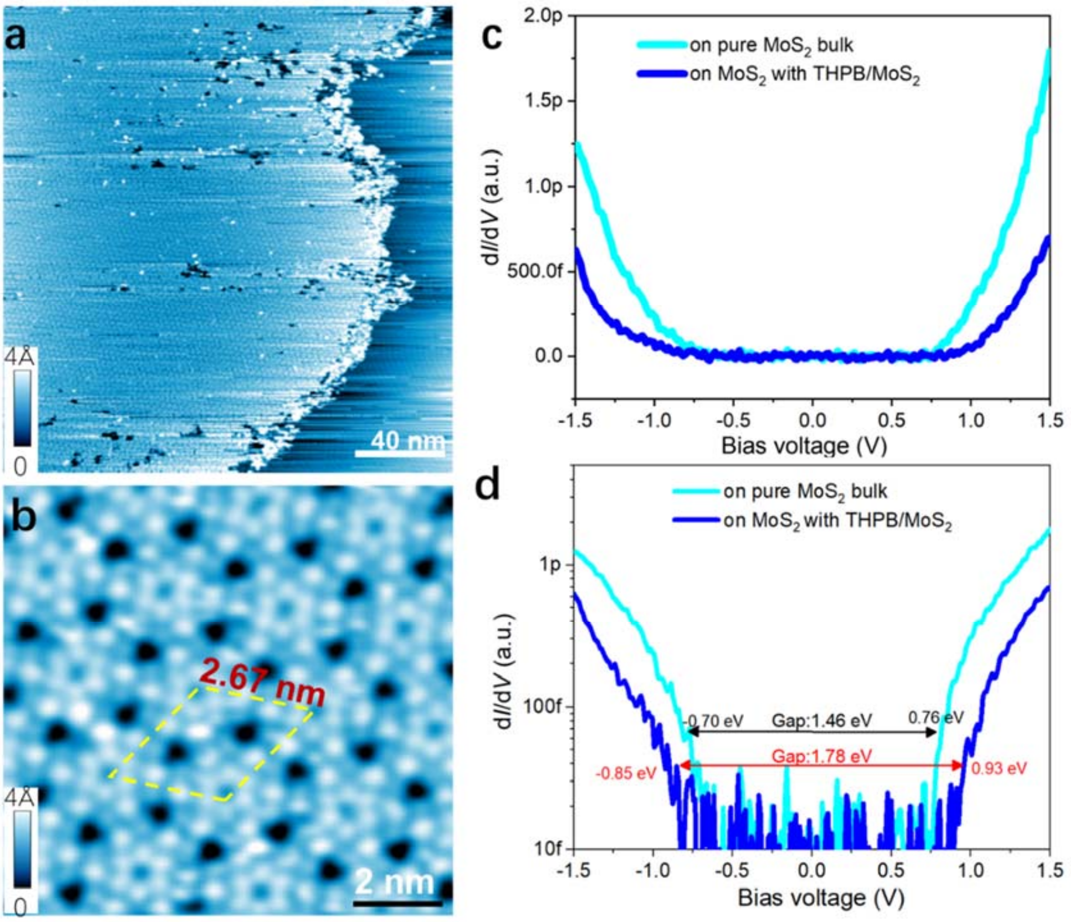

Supplementary Fig. 12 | High resolution STM images and dI/dV tunneling spectroscopic measurements on THPB on MoS_2. a, large scale image show THPB island formed on vacuum-cleaved MoS_2 surface. The image is 200×200 nm^2 with the setting parameters of V_B: -5V and I_T:5pA. **b**, high-resolution image of THPB framework on MoS_2.The image are 10×10 nm^2 with the setting parameters of V_B:-5V and I_T:5pA. The image shows similar structure as the THPB-HOF on HOPG, the size of unit cell is about 2.67 nm, larger than the size of unit cell of THPB-frame on HOPG (16.5 Å). **c-d**, dI/dV spectrum measured on clean MoS_2 surface (cyan) and THPB framework (blue), respectively, **c,** data plotted as linear coordination and **d,** data plotted as log coordination. Actually, the bandgap of MoS_2 clean surface is measured about 1.46 eV, smaller than the bandgap (1.78 eV) measured on THPB-framework, indicating the self-lifting effect of THPB-framework applying on the MoS_2 layer. Our results are consistence with the reported bandgap transition of MoS_2 from bulk to monolayer. This result provides the evidence of the universality of our method.

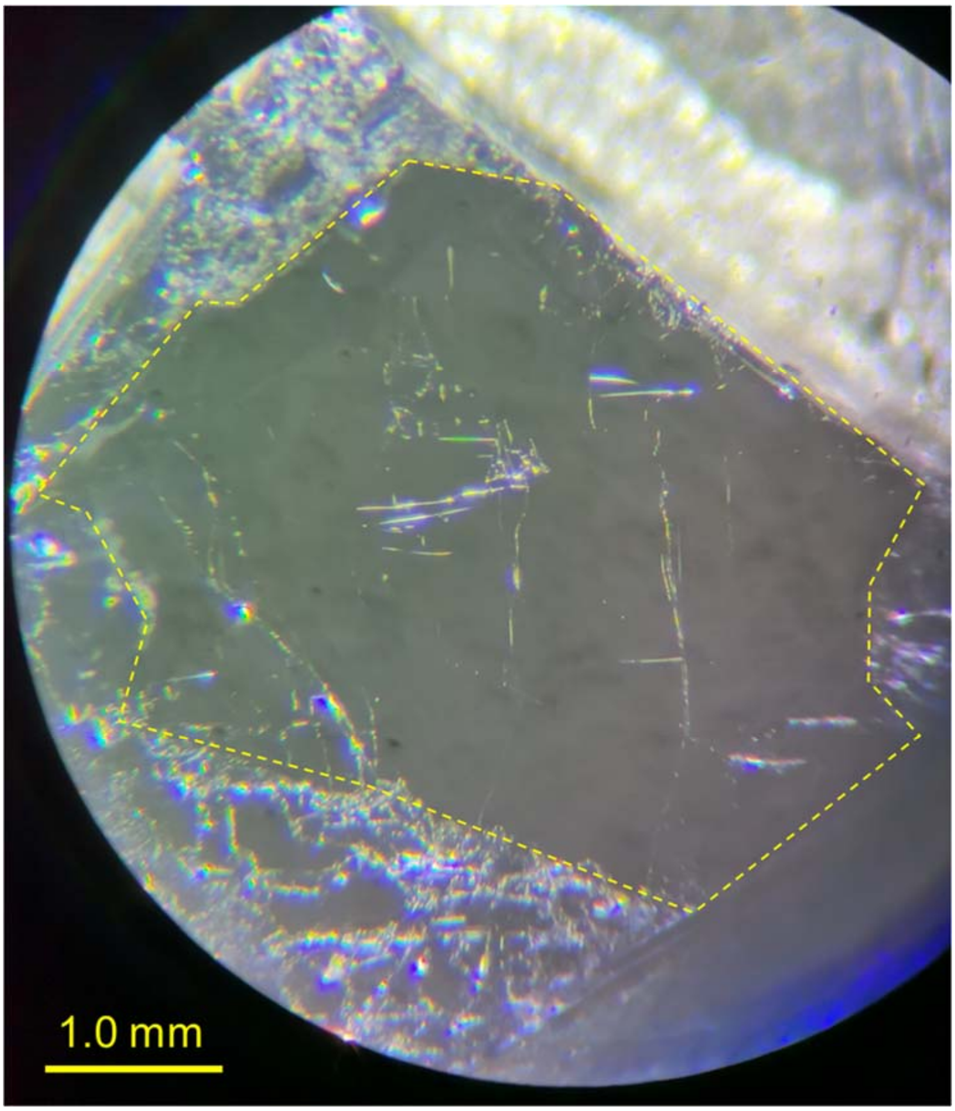

Supplementary Fig. 13 | The optical image of HOPG sample. HOPG surface is obtained via vacuum cleavage. As we see from the image, the flat surface area of HOPG marked by yellow dashed lines is about 2x3 mm^2. Within such area, the uniform THPB-HOF could form.

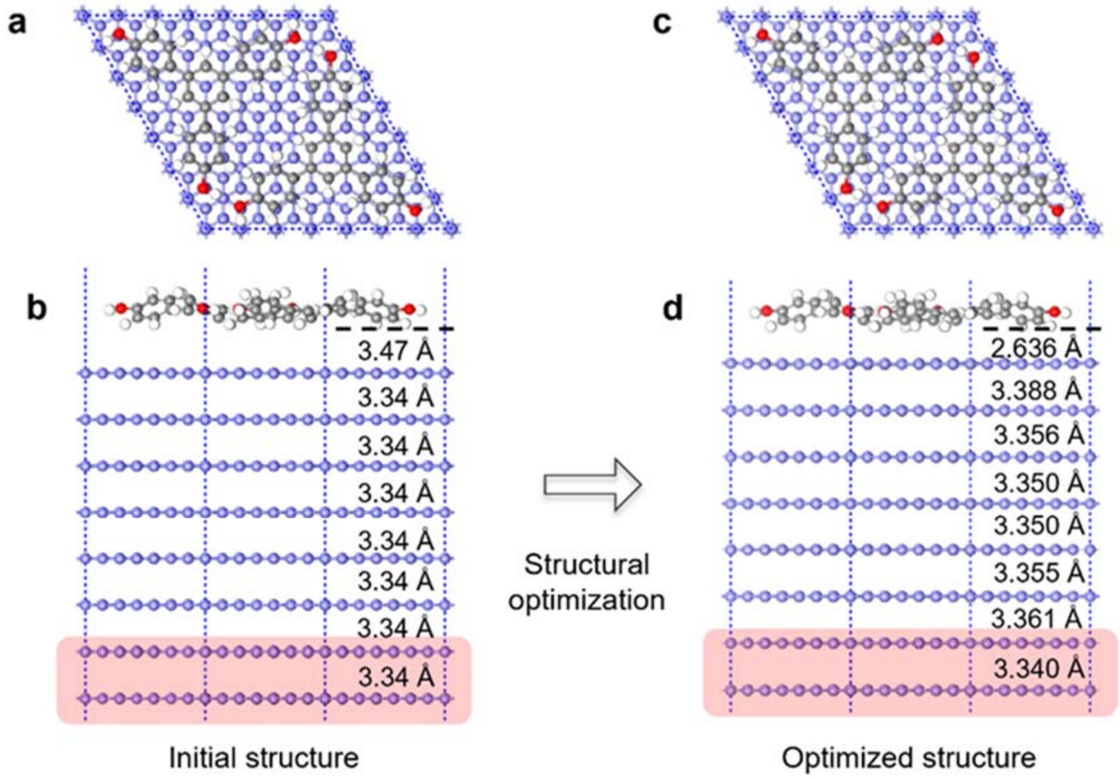

Supplementary Fig. 14 | Structural relaxation of the THPB-HOF monolayer on top of the eight-layered HOPG substrate. a-b, top and side view of the initial structure. **c-d,** same as a-b but for the final optimized structure. Here the atomic positions of the bottom two layers of HOPG (highlighted by the pink shadow) are fixed and all other atomic positions are relaxed during the structural optimization calculation, to mimicking an infinite thick HOPG substrate meanwhile reducing the computational cost.

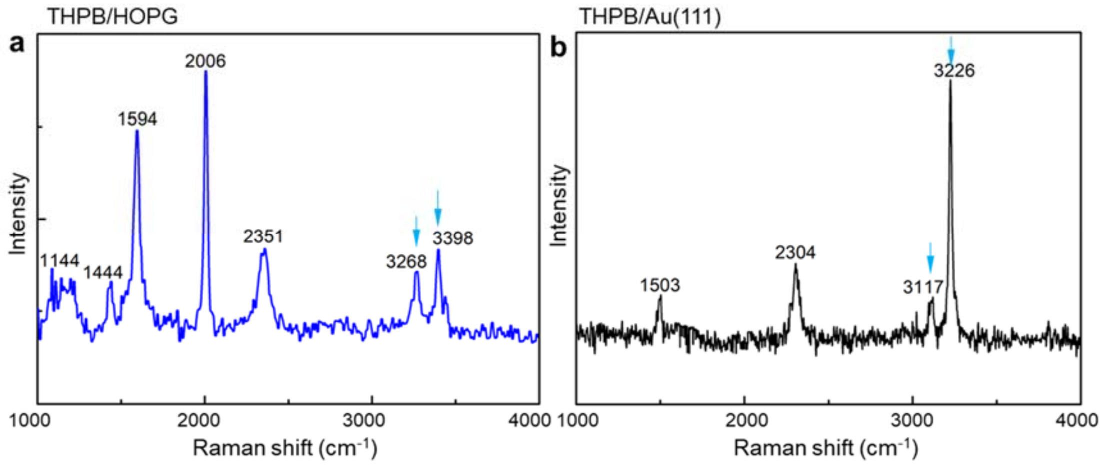

Supplementary Fig. 15 | In-situ room-temperature Raman spectrum from 1000 cm^{-1} to 4000 cm^{-1} for THPB-HOF/HOPG **(a)** and THPB-HOF/Au(111) **(b)** for comparison. Two characteristic Raman peaks (3268 and 3398 cm^{-1}), similar to the modes (3117 and 3226 cm^{-1}) on Au(111), can be assigned to the H-O vibration [11], indicating the strength of H-bond similar to that in water at ≈ 3400 cm^{-1} [12]. Besides, the mode of 1594 cm^{-1} can be assigned to the *G* peak of graphene (≈1588 cm^{-1}) [13] with a upshift of 6 cm^{-1}. Note, the *2D* peak of graphene (≈ 2689 cm^{-1}) disappear, may owing to the strong interlayer interaction between THPB-HOF and graphene.

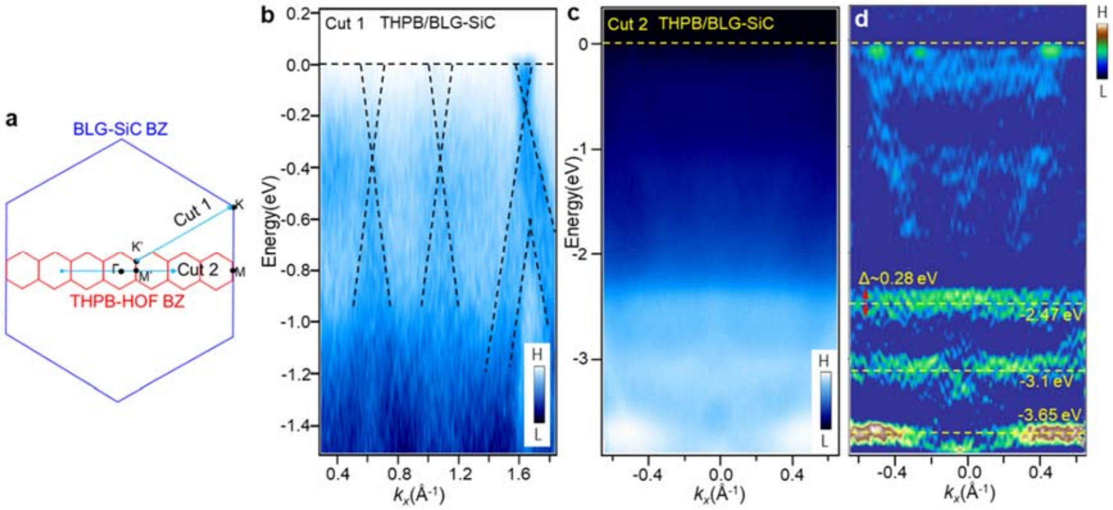

Supplementary Fig. 16 | ARPES observation of the THPB/BLG-SiC bands. a, The relationship between the BZs of BLG-SiC and THPB-HOF. The cyan-dashed lines indicate the paths of Cut 1 and 2, taken for measurement in **b**-**d**. **b**, ARPES intensity plot measured along the path of cut 1 from -1.5 eV to E_F. A series of linear dispersive bands can be clearly resolved, especially a dual Dirac cones at the BZ boundary, corresponding to the splitting of the π state in the BLG epitaxially-grown on SiC [[14,15]]. Other linear bands are Dirac bands of BLG folded into the BZ of THPB. **c**-**d,** High-resolution ARPES spectra **(c)** and second-derivative intensity plot **(d)**, taken along the path of cut 2, taken from -3.85 eV to 0 eV. Three non-dispersive bands can be clearly resolved in (**d**) at the energies of -2.47, -3.1 and -3.65 eV with the bandwidth about 0.28 eV. Again, these narrow bands can be the bands of THPB-HOF.

Supplementary references:

1. Cleland, W. W., and Maurice M. Kreevoy. Low-Barrier Hydrogen Bonds and Enzymic Catalysis. *Science* **264**, 1887-1890(1994).
2. S.Y. Zhou, G.H. Gweon, C.D. Spataru, J. Graf, D.-H. Lee, Steven G. Louie, A. Lanzara, *Phys. Rev. B* **71**, 161403(R) (2005).
3. R. Kundu, P. Mishra, B.R. Sekhar, M. Maniraj, S.R. Barman, Electronic structure of single crystal and highly oriented pyrolytic graphite from ARPES and KRIPES, *Physica B* **407**, 827–832(2012).
4. T. Kihlgren, T. Balasubramanian, L. Wallde´n, R. Yakimova, Narrow photoemission lines from graphite valence states. *Phys. Rev. B* **66**, 235422 (2002).
5. K. Sugawara, T. Sato, S. Souma, T. Takahashi, H. Suematsu, Fermi surface and edge-localized states in graphite studied by high-resolution angle-resolved photoemission spectroscopy. *Phys. Rev. B* **73**, 045124 (2006).
6. J.-C. Charlier, X. Gonze, J.-P. Michenaud, First-principles study of the electronic properties of graphite. *Phys. Rev. B* **43**, 4579(1991).
7. R.F. Willis, B. Fitton, G.S. Painter, Secondary-electron emission spectroscopy and the observation of high-energy excited states in graphite: Theory and experiment. *Phys. Rev. B* **9**, 1926(1974).
8. N.A.W. Holzwarth, S.G. Louie, S. Rabii, X-ray form factors and the electronic structure of graphite. *Phys. Rev. B* **26**, 5382(1982).
9. R.C. Tatar, S. Rabii, Electronic properties of graphite: A unified theoretical study. *Phys. Rev. B* **25**, 4126(1982).
10. Nikhil Sivadas, Satoshi Okamoto, and Di Xiao, Gate-Controllable Magneto-optic Kerr Effect in Layered Collinear Antiferromagnets, *Phys. Rev. Lett.* **117**, 267203 (2016)
11. Antonela C. Marele, *et al.* Some Pictures of Alcoholic Dancing: From Simple to Complex Hydrogen-Bonded Networks Based on Polyalcohols, *J. Phys. Chem. C*, **117**,4680-4690 (2013).
12. Seki, T. *et al.* The Bending Mode of Water: A Powerful Probe for Hydrogen Bond Structure of Aqueous Systems. *J. Phys. Chem. Lett.* **11**, 8459-8469 (2020).

13. Lv, R. T. *et al.* Nitrogen-doped graphene from atmospheric-pressure CVD: beyond single substitution and enhanced molecular sensing”, *Sci. Rep.* **2**, 586(2012)

14. Wang, C. *et al.* Direct Observation of Global Elastic Intervalley Scattering Induced by Impurities on Graphene. *Nano Lett.* **21**, 19, 8258–8265 (2021)

15. Ohta, T.; Bostwick, A.; Seyller, T.; Horn, K.; Rotenberg, E. Controlling the electronic structure of bilayer graphene. *Science* **313**, 951–954 (2006).
