# Supplementary material for: Large-scale 2D heterostructures from hydrogen-bonded organic frameworks and graphene with distinct Dirac and flat bands
Source: Nat Commun. 2024 Jul 15;15:5934. doi: 10.1038/s41467-024-50211-5 (PMC11250822; doi:10.1038/s41467-024-50211-5)
Supplement: Supplementary file 3 — Description of Additional Supplementary Files [file 41467_2024_50211_MOESM3_ESM.pdf]

Description of Additional Supplementary Files

File Name: Supplementary Data 1

Description: An optimized structural file of the heterostructure including a molecular monolayer and eight layers of HOPG.
